# Selective Deposition of Hard Boron-Carbon Microstructures on Silicon

**DOI:** 10.3390/ma14061397

**Published:** 2021-03-13

**Authors:** Gopi Samudrala, Kallol Chakrabarty, Paul A. Baker, Bernabe S. Tucker, Yogesh K. Vohra, Shane A. Catledge

**Affiliations:** 1 Department of Physics, University of Alabama at Birmingham, Birmingham, AL 35294, USA; kallol89@uab.edu (K.C.); pabaker@uab.edu (P.A.B.); ykvohra@uab.edu (Y.K.V.); catledge@uab.edu (S.A.C.); 2 Department of Materials Science & Engineering, University of Alabama at Birmingham, Birmingham, AL 35294, USA; betucker@uab.edu

**Keywords:** maskless lithography, chemical vapor deposition, ultrahard materials, microstructures, nanoindentation, MEMS

## Abstract

Boron-rich B-C compounds with high hardness have been recently synthesized by the chemical vapor deposition (CVD) method. In this paper, we present our successful efforts in the selective growth of microstructures of boron-carbon compounds on silicon substrates. This was achieved by combining microfabrication techniques such as maskless lithography and sputter deposition with the CVD technique. Our characterization studies on these B-C microstructures showed that they maintain structural and mechanical properties similar to that of their thin-film counterparts. The methodology presented here paves the way for the development of microstructures for microelectromechanical system (MEMS) applications which require custom hardness and strength properties. These hard B-C microstructures are an excellent choice as support structures in MEMS-based devices.

## 1. Introduction

Boron-rich B-C compounds, many of which are high-temperature ceramic materials, are utilized in application areas where hard materials are needed. The excellent thermal stability and high mechanical strength offered by these materials are desirable for applications in extreme conditions of pressure and temperature. These compounds are synthesized by techniques ranging from carbothermal reduction [1] for powders to chemical vapor deposition [2] for whiskers and platelets. However, recent advancements in combining first-principles studies with experimental methods allowed us to deposit crystalline B_50_C_2_ thin films by the microwave plasma chemical vapor deposition method [3]. We showed successful deposition of these thin films—with the hardness of 37 GPa—on silicon substrates. Further advancements in this technique allowed us to deposit structural variations of these coatings with a controlled degree of crystallinity, yielding measured hardness values of 64 GPa [4].

While different stoichiometric boron carbides have found uses in areas such as wear-resistant components, cutting tools and neutron absorbers [5,6,7,8], a hitherto unexplored area is the development of B-C devices using microfabrication technologies including lithography. A problem in achieving this goal has been the difficulty in producing controlled micro or nano-scale structures based on these B-C compounds. While nanocrystalline powders of boron carbide compounds have been produced by various methods [1,9], they cannot be synthesized as controlled micro-patterned structures to exploit the benefits of this material’s physical properties for device-based applications. A point to note is that most B-C compounds with an extremely high atomic percentage of boron (like B_50_C_2_) are insulators. However, some high-temperature boron carbides are semiconductors [10,11]. 

Our present work addressed this opportunity by producing well-controlled micro-patterns of B_50_C_2_ which we have previously produced in the thin-film form [3,4]. Microfabrication techniques such as maskless lithography and sputter deposition are very effective tools for selective area masking. By combining the microfabrication techniques with chemical vapor deposition (CVD), we successfully grew microstructures of B_50_C_2_ on silicon substrates. 

Development of microstructures with tunable material properties to suit a particular application area is a topic of great interest for researchers. A wide variety of materials are being investigated to achieve this. Some examples include development of glassy carbon microelectromechanical systems (MEMS) with tunable hardness [12] and nanotwinned metallic MEMS structures with high strength [13], achieving tunability in mechanical properties of silicon oxynitride for MEMS-based detectors [14]. As such, the need for support structures that have high strength and hardness in the rapidly evolving landscape of MEMS device designs can be easily anticipated. One such MEMS device application would involve space missions. With increasingly complex missions in extreme environments comes the need for on-board devices that can withstand a wide range of temperatures and pressures [15,16]. In this paper, we demonstrate for the first time the feasibility of selective area deposition of hard B-C microstructures on silicon and potential utility of these structures in MEMS devices. 

## 2. Materials and Methods

The silicon substrates used in our experiments were N-type (phosphorus-doped) with <100> orientation (University wafer item#736). Dimensions of substrates used for B-C microstructures’ growth were 5 mm × 5 mm with a thickness of 0.28 mm. The growth of B-C microstructures was achieved by combining microfabrication techniques with CVD. A pre-defined pattern was transferred onto the silicon substrate with maskless lithography. The metal mask on silicon substrate was applied and etched at appropriate times to achieve growth of B-C microstructures. Details of the lithography process are discussed below.

### 2.1. Lithography Process

**Figure 1:**
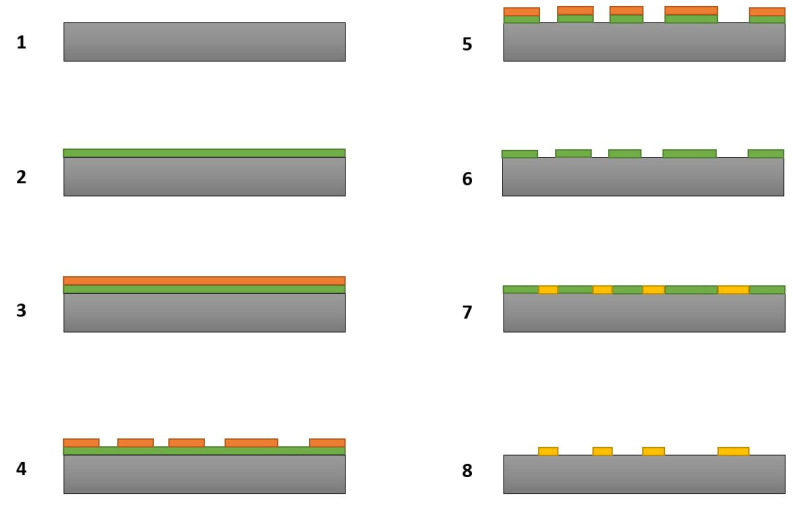
Steps involved in the fabrication of B-C microstructures: (**1**) Silicon substrate, (**2**) Sputter deposition of tungsten mask, (**3**) Application of photoresist, (**4**) Transfer of pattern onto photoresist, (**5**) Etching of selective areas of tungsten mask, (**6**) Removal of residual photoresist, (**7**) Chemical vapor deposition (CVD) of B-C compound in selective areas, (**8**) Etching of remaining tungsten mask to obtain B-C microstructures.

We employed maskless lithography to transfer different types of microstructures onto silicon substrates. A benefit of this type of lithography is that it allows users to transfer complex patterns onto the substrates without the use of physical masks. Figure 1 shows in detail all the steps involved in the fabrication of the B_50_C_2_ microstructures. The first step is the creation of a mask on the substrate. The requirement here is that the mask should survive the harsh CVD plasma environment in which the B-C microstructures will be grown. Tungsten was deliberately chosen for the mask material. As we have shown in our previous works [17,18], tungsten survives well in the harsh CVD plasma conditions. After depositing a tungsten thin film, the substrate was then transferred to the maskless photolithography system where our desired pattern was then “printed” on the substrate. A positive tone photoresist (Microposit SC1827) and AZ-300 developer were used in the lithography process. By etching selected areas of the tungsten mask, we exposed silicon where the B-C compound could be grown. A commercially available chemical etchant (product #667498, Sigma-Aldrich, St. Louis, MO, USA) was used for dissolving tungsten masks after necessary steps were performed in our experiments. After CVD processing of the substrate, a second round of wet etching removed the remaining tungsten mask to reveal as grown B-C microstructures. An even more detailed explanation for the lithography process and how we employed it in different application areas can be found in our previous publications [17,18]. 

### 2.2. MPCVD Process

**Figure 2:**
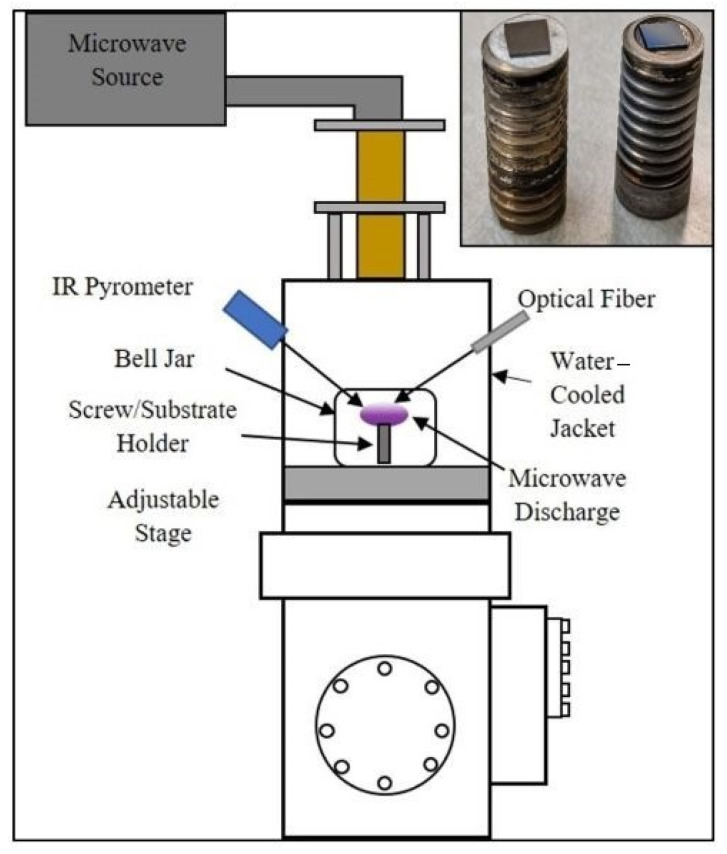
Schematic of the 6 kW microwave plasma chemical vapor deposition (MPCVD) chamber used in our experiments. The inset shows the two types of substrate holders utilized in the CVD growth of B-C microstructures.

Boron-rich boron carbide coatings were grown in a microwave plasma chemical vapor deposition (MPCVD) system (Wavemat Inc. Plymouth, MI, USA) shown in Figure 2. The sample surface is heated by direct contact with the plasma. The substrate holder and the outer resonance cavity jacket on the MPCVD reactor are water-cooled. A quartz bell jar isolates the low-pressure plasma environment from the resonance cavity. N-type <100> orientation silicon substrates with the desired lithographic pattern were placed on the surface of a 0.5” diameter molybdenum screw (Figure 2). Microwave power of 1 kW was used for growth experiments. Chamber pressure was 15 Torr during the deposition process. Hydrogen (H_2_) was used as the carrier gas and a diborane mixture (90% H_2_, 5% B_2_H_6_, with 5 ppm methane in B_2_H_6_ gas acting as the source of carbon) as the reactive gas. The gas flow rates were 500 standard cubic centimeters per minute (SCCM) of hydrogen and 2 SCCM of the diborane mixture. Total deposition time was 6 h with an average substrate temperature of 800 °C. 

### 2.3. Characterization Techniques

The samples were examined by scanning electron microscopy (SEM) and X-ray photoelectron spectroscopy (XPS) to confirm that the as-grown B-C patterns retained the microstructure and crystallinity of their thin-film counterparts. Nanoindentation studies were carried out using an MTS NanoIndenter G200 with a Berkovich diamond tip (nominal radius 50 nm). A fused silica standard with accepted Young’s modulus value of 72 GPa was tested before and after indenting our samples. The range of Young’s modulus of this silica standard was consistent with the accepted value. Hence, we could confirm the preservation of tip geometry during the sample testing. All indents, including those on silica, were made to a maximum depth of 600 nm. The measured hardness was determined at maximum load. 

SEM analysis with a Quanta FEG Instrument (by FEI/Thermo Fisher) was accomplished by attaching the silicon substrates to aluminum sample studs with high vacuum adhesive. Further, strips of aluminum tape were added to ensure good grounding, and the high vacuum mode was selected. The samples were analyzed with an accelerating voltage of 20 kV and the spot size (non-specified units, unique to the instrument) set to 3.0, and slow scan speeds around 100 microseconds were used for image capture. A variety of magnifications were used to elucidate the microstructure of the B-C structures on the silicon substrates. The XPS instrumentation was a Phi Electronics Versaprobe 5000 (Phi Electronics, Chanhassen, MN, USA), equipped with a micro-focused Al monochromatic source (λ = 1486.6 eV) and a dual anode conventional X-ray source with a neutralizer. Survey spectra were taken with an incident energy of 1253.6 eV, and both sources were used for data collection. 

Characterization of microstructures with Raman spectroscopy was carried out using a Dilor XY Modular spectrometer. The spectrometer was equipped with a liquid nitrogen-cooled Princeton Instruments Acton detector. The software accompanying the detector (Winspec32) was used to record the data in the range of 400–1800 cm^−1^. A 1200 grooves/mm grating on the spectrometer and a 100X microscope objective were utilized to gather the data. A 200 mW frequency-doubled YAG laser was the excitation source. The laser wavelength is 532 nm. 

## 3. Results

**Figure 3:**
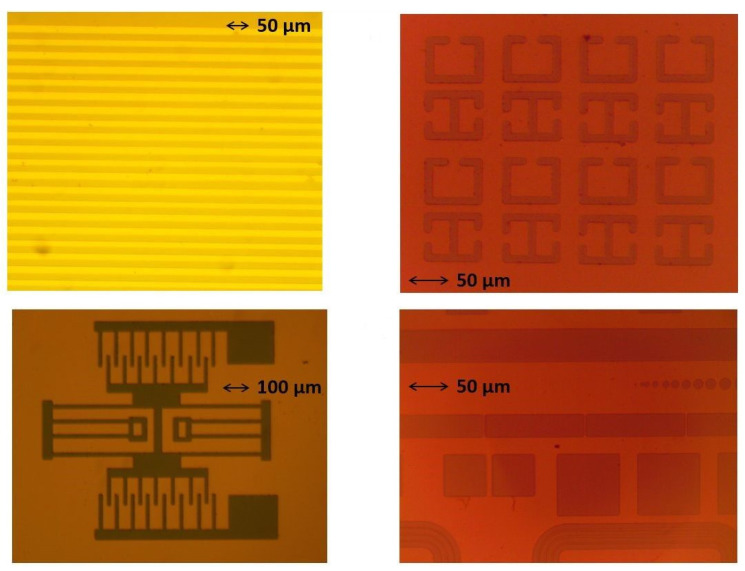
Various designs transferred onto the tungsten mask on silicon substrates. These images correspond to step 6 in Figure 1.

We were able to successfully combine microfabrication techniques and CVD to produce boron-carbon microstructures that have dimensions as small as 10 microns. A diverse set of shapes inspired by MEMS designs, such as radio frequency (RF) ring resonator structures, were transferred onto silicon substrates. Figure 3 shows various designs we “printed” on silicon substrates. These images correspond to step 6 in Figure 1. We successfully managed to grow boron-rich B-C microstructures in all these geometries. 

**Figure 4:**
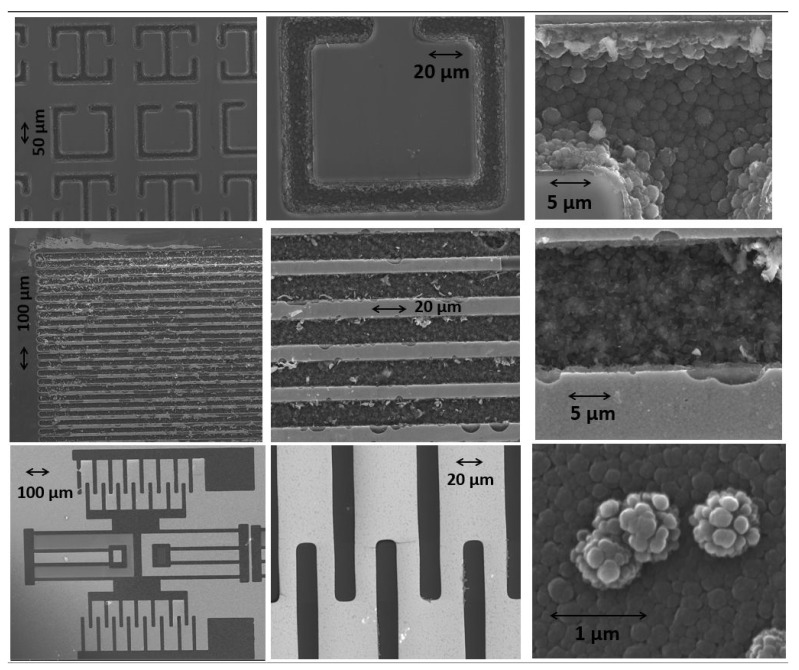
SEM images of B-C microstructures grown on silicon substrates. The shapes in the top row are inspired by radio frequency (RF) ring resonator structures. The middle row shows a densely packed grid of B-C microstructures with a gap of 10 microns between the lines. The bottom row shows B-C microstructures in the geometry of a MEMS resonator.

SEM imaging of the B-C microstructures revealed a similar crystalline structure as observed from the thin-film form of the B-C material [3]. Figure 4 shows the SEM images from various samples we grew. The top row shows a grid of microstructures whose design was inspired by the RF ring resonator structures. In the middle row, a compact grid of B_50_C_2_ microstructures is displayed. Well-controlled patterns with features as small as 10 microns in width were reliably grown on the silicon substrates. The bottom row shows the SEM images of the B-C microstructures which were grown in the geometry of a typical MEMS resonator design. 

Determination of crystal structure of these as-grown B-C microstructures through X-ray diffraction is very difficult. Because these microstructures cover a very small portion of the silicon substrate surface (less than 20% surface area), determining crystalline structure through the use of a typical lab-scale XRD system is not feasible. We thus relied on Raman spectroscopy and XPS to verify that these microstructures are indeed boron-rich B-C compounds. In addition to the data presented below, energy dispersive spectroscopy (EDS) during our SEM analysis also showed the presence of a high atomic percentage of boron in these microstructures. 

**Figure 5:**
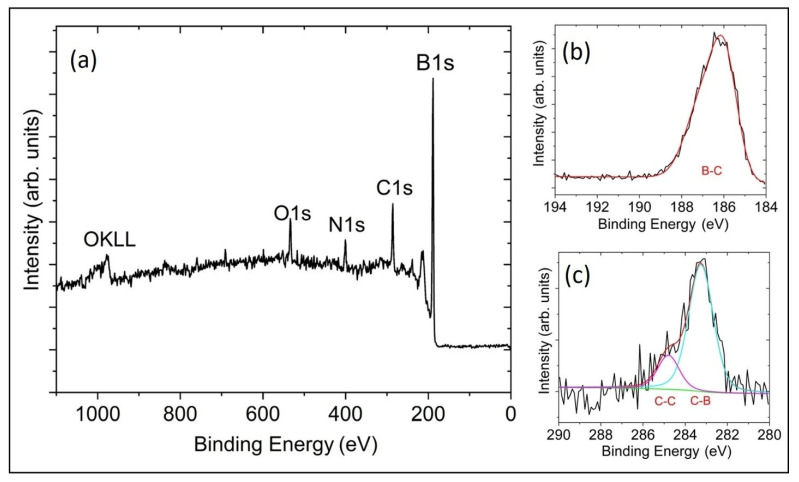
X-ray photoelectron spectroscopy survey scans of (**a**) the B-C microstructure with an elemental composition of 80.6% B, 12.4% C, 3.8% of N and 3.2% O. Panels (**b**,**c**) show high-resolution scans for B1s and C1s, respectively, with corresponding peaks assigned to B-C, C-B and C-C (adventitious carbon) bonding.

The XPS of the B-C microstructure sample showed that the surface is composed of 80.6% B, 12.4% C, 3.8% of N and 3.2% O (rel. at%) with no other elements present. The small amount of oxygen contamination seen on the sample surface is generally present in samples that were exposed to air. Table 1 shows the complete peak assignments with corresponding binding energy. The high-resolution B1s scan in Figure 5b shows that 100% of the boron is B-C-bonded. The high-resolution C1s scan in Figure 5c shows that 20% of the carbon is C-C-bonded and the remaining 80% is B-C-bonded [19,20]. Using this information, our XPS measured carbon content in the B-C-bonded BC microstructure sample is 9.9%. This result is comparable to our previous study of B_50_C_2_ coating by microwave plasma CVD [3,4].

**Figure 6:**
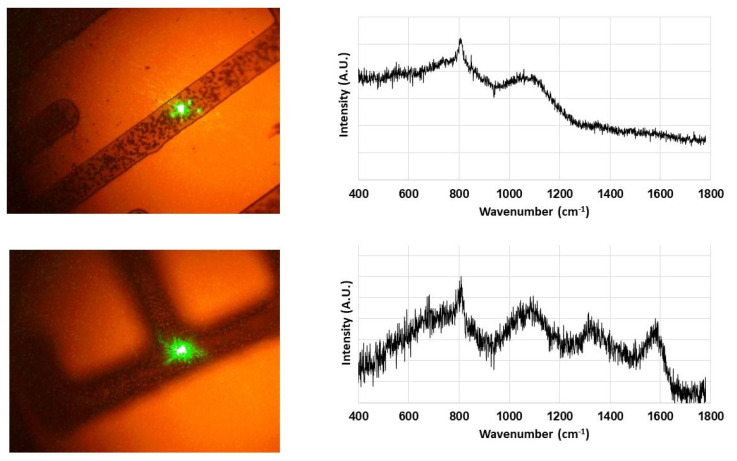
Raman spectra of B-C microstructures. The green dot on the microstructures is the 532 nm laser used for spectroscopy. Raman spectra show the presence of both amorphous (top row) and crystalline B_50_C_2_ (bottom row) in these microstructures.

Raman spectroscopy was performed on these B-C microstructures. The results shown in Figure 6 are again in good agreement with our previous results [3,4]. The 532 nm laser used for spectroscopic studies was carefully positioned on the grown B-C microstructures as shown in Figure 6. The characteristic broad peaks indicating the presence of amorphous structures were observed from the sample in the top row of Figure 6. These are the bands centered around 800 cm^−1^ and 1100 cm^−1^ which have been attributed to the B_4_C structural framework [21]. In addition to these two bands, the two broad bands centered around 1340 cm^−1^ and 1570 cm^−1^, commonly known as “D” and “G” bands, reveal the presence of disordered carbon (bottom row). The presence of these disordered carbon bands has also been attributed to carbon in boron carbide materials in literature [4,6,21]. Combined with the similarity in XPS results from the thin-film counterparts of these B-C microstructures, they can indeed be characterized as B_50_C_2_. 

Some samples showed both types of Raman signatures at different points of the microstructures. This leads us to conclude that these microstructures contain a mixture of amorphous and crystalline B_50_C_2_. This is in contrast to our previous study [4] where we achieved control over the degree of crystallinity by using two different types of substrate holders (Figure 2 inset). The presence of tungsten mask on the substrates in this study adds a further layer of separation of sample surface from the CVD plasma. This could be the reason for the loss of control over the degree of crystallinity in the CVD-grown material. 

**Figure 7:**
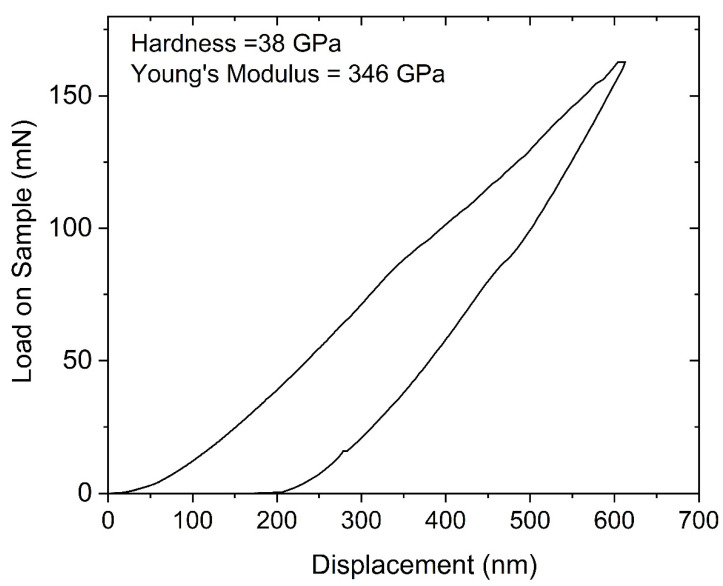
Nanoindentation load-displacement curve from one location on the boron-rich boron carbide microstructure to a depth of 600 nm. The extracted values of nanoindentation hardness and Young’s modulus are also indicated.

Figure 7 shows nanoindentation load/displacement data of the boron-rich boron carbide microstructure coating. The indent with the highest measured hardness was 38 GPa and had Young’s modulus of 346 GPa. The relative contribution of elastic and plastic deformation can be calculated from the final unloading depth of the load-displacement curves. A high elastic recovery (about 67%) from the boron carbide coating is measured from the unloading data. The hardness value of the boron-rich boron carbide microstructure was comparable with the B_50_C_2_ thin film deposited by the CVD technique [3].

## 4. Conclusions

The maskless lithography technique was combined with the microwave plasma chemical vapor deposition for the first time in the growth of hard B-C microstructures on silicon substrates. The measured nanoindentation hardness, Raman and X-ray photoelectron spectroscopies carried out on B-C microstructures are in close agreement with our earlier data on B_50_C_2_ thin films. Machine learning algorithms are now being utilized to predict the metastable phases of B-C-N compounds which are superhard and have high thermal stability [22]. A roadmap for developing microstructures for MEMS applications that require custom hardness and strength properties could proceed as follows: *determine material property needs for a given application → identify suitable materials through first-principles calculations or machine learning → develop materials through suitable techniques → combine said technique with appropriate microfabrication processes to produce microstructures.* The results presented in this paper show the feasibility of combining maskless lithography with chemical vapor deposition to create novel superhard microstructures for extreme environments.

## Figures and Tables

**Table 1:** XPS compositional analysis and fitted parameters of B1s and C1s. [19,20].

Sample	Peaks	Binding Energy	Peak Area (%)	Assignment
B-C Microstructure	B1s	186.5	100	B-C
	C1s	283.3	80	C-B
	C1s	284.7	20	C-C

## Data Availability

The data presented in this study are available on request from the corresponding author. The data are not publicly available due to privacy reason.
